# Copper-Catalyzed Glutathione Oxidation is Accelerated by the Anticancer Thiosemicarbazone Dp44mT and Further Boosted at Lower pH

**DOI:** 10.1021/jacs.2c05355

**Published:** 2022-08-05

**Authors:** Enrico Falcone, Alessandra G. Ritacca, Sonja Hager, Hemma Schueffl, Bertrand Vileno, Youssef El Khoury, Petra Hellwig, Christian R. Kowol, Petra Heffeter, Emilia Sicilia, Peter Faller

**Affiliations:** † Institut de Chimie (UMR 7177), University of Strasbourg − CNRS, 4 Rue Blaise Pascal, 67081 Strasbourg, France; ‡ Department of Chemistry and Chemical Technologies, Università della Calabria, Ponte P. Bucci, 87036 Arcavacata di Rende, (CS), Italy; § Center for Cancer Research, Medical University of Vienna, Borschkegasse 8a, 1090 Vienna, Austria; ∥ Laboratoire de bioélectrochimie et spectroscopie, UMR 7140, CNRS, Université de Strasbourg, 4 Rue Blaise Pascal, 67081 Strasbourg, France; ⊥ Institute of Inorganic Chemistry, Faculty of Chemistry, University of Vienna, Waehringer Straße 42, 1090 Vienna, Austria; # Institut Universitaire de France (IUF), 1 rue Descartes, 75231 Paris, France

## Abstract

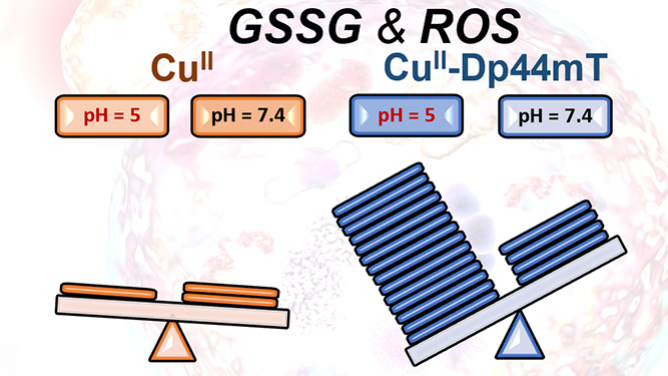

Glutathione (GSH) is the most abundant thiol in mammalian cells and plays a crucial role in maintaining redox cellular homeostasis. The thiols of two GSH molecules can be oxidized to the disulfide GSSG. The cytosolic GSH/GSSG ratio is very high (>100), and its reduction can lead to apoptosis or necrosis, which are of interest in cancer research. Cu^II^ ions are very efficient oxidants of thiols, but with an excess of GSH, Cu^I^*_n_*(GS)*_m_* clusters are formed, in which Cu^I^ is very slowly reoxidized by O_2_ at pH 7.4 and even more slowly at lower pH. Here, the aerobic oxidation of GSH by Cu^II^ was investigated at different pH values in the presence of the anticancer thiosemicarbazone Dp44mT, which accumulates in lysosomes and induces lysosomal membrane permeabilization in a Cu-dependent manner. The results showed that Cu^II^-Dp44mT catalyzes GSH oxidation faster than Cu^II^ alone at pH 7.4 and hence accelerates the production of very reactive hydroxyl radicals. Moreover, GSH oxidation and hydroxyl radical production by Cu^II^-Dp44mT were accelerated at the acidic pH found in lysosomes. To decipher this unusually faster thiol oxidation at lower pH, density functional theory (DFT) calculations, electrochemical and spectroscopic studies were performed. The results suggest that the acceleration is due to the protonation of Cu^II^-Dp44mT on the hydrazinic nitrogen, which favors the rate-limiting reduction step without subsequent dissociation of the Cu^I^ intermediate. Furthermore, preliminary biological studies in cell culture using the proton pump inhibitor bafilomycin A1 indicated that the lysosomal pH plays a role in the activity of Cu^II^-Dp44mT.

## Introduction

**Chart 1:**
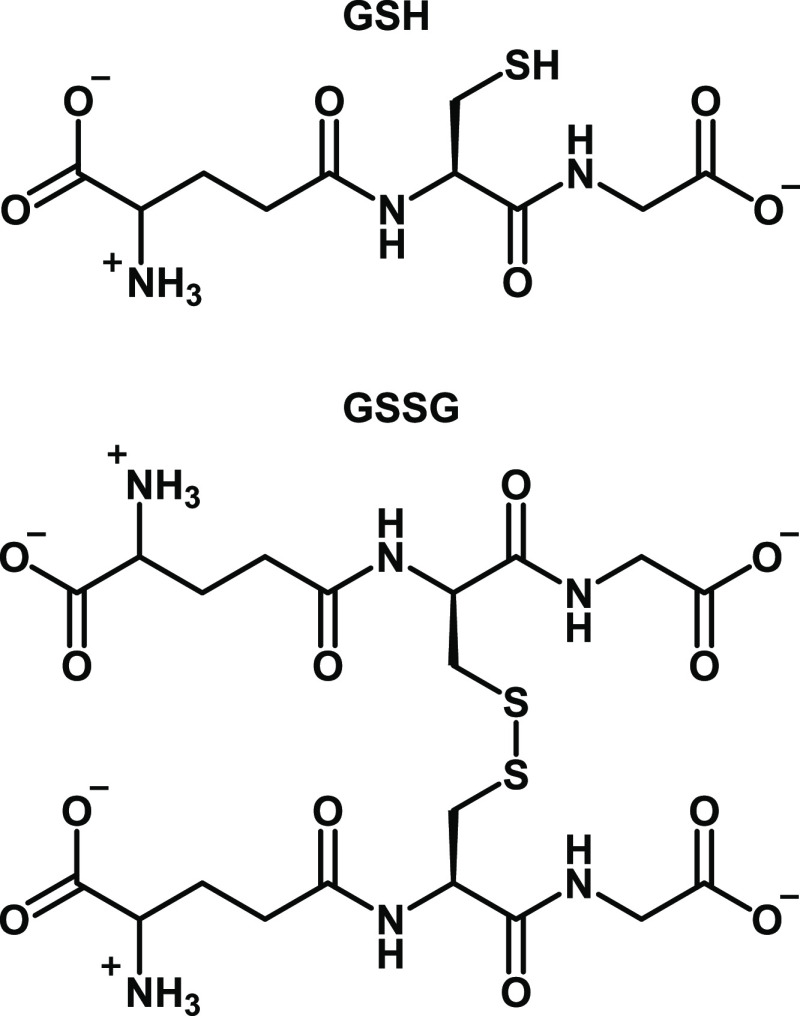
Structure of Reduced Glutathione (GSH) and Oxidized Glutathione Disulfide (GSSG)

Glutathione (γ-glutamyl-cysteinyl-glycine, GSH, see Chart 1) is one of the most concentrated biomolecules (0.5–15 mM) and the most abundant low-molecular-weight thiol in mammalian cells.

The reduced/oxidized glutathione (GSH/GSSG) couple is the main intracellular regulator of redox homeostasis in animals and plants. Under physiological conditions, most GSH is present in the reduced state, exceeding a GSH/GSSG ratio of 1000:1 in cytosol and nucleus, whereas a shift to a lower value of this ratio leads to apoptosis or necrosis.^1^ A lower GSH/GSSG ratio (∼3:1) is also physiologically found in the endoplasmic reticulum (ER) and the secretory pathway.^2^

GSH has several important roles: control of the thiol oxidation state of proteins, defense against oxidative stress, and detoxification. It also acts as an antioxidant, both by directly scavenging reactive oxygen species (ROS) and by repairing their damage via enzymatic processes. Thus, GSH is a crucial compound for living cells, and targeting GSH metabolism is of wide interest for therapeutic purposes, in particular for fighting cancer.^3^ Indeed, cancer cells often have high GSH concentrations, and elevated GSH levels are indicative of tumor progression and increased drug resistance.^4^ Depletion of GSH, then, is considered to be a promising anticancer strategy, in particular in combinatorial approaches.^5^

In general, thiols can be oxidized to disulfides under aerobic conditions according to the following reaction
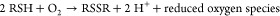
iUnder aerobic conditions, this is a spontaneous reaction, and the cystine/cysteine couple has a quite low standard redox potential of about −0.22 V at pH 7 vs SHE,^6^ while for the GSSG/GSH couple, it is slightly lower, −0.26 V,^1^ at the same pH. According to the Nernst equation, the redox potential increases by ∼59 mV per electron exchanged and per pH unit below p*K*_a_. Hence, reaction i becomes more and more thermodynamically unfavorable as the pH decreases. Besides the thermodynamic driving force, the kinetics of thiol oxidation, as well as the so-called thiol–disulfide exchange (the redox reaction between thiols and disulfides), is also slowed down by lowering the pH. Thus, lowering the pH is an approach used to quench thiol oxidation and disulfide exchange reactions. The lower reactivity of thiols at lower pH is generally attributed to the significantly higher reactivity of thiolates (i.e., deprotonated thiols, RS^–^) compared to thiols. Thiolates are, indeed, better nucleophiles and, hence, react quickly with electrophiles like disulfide bonds, O_2_, H_2_O_2_, other ROS, metal ions, etc. Whereas some thiols react rapidly, GSH oxidation is quite sluggish at pH 7 and the activity drops notably by lowering the pH.^7^ Moreover, the p*K*_a_ value of a thiol is crucial in determining its reactivity, as demonstrated by the higher reactivity of cysteine (Cys) (p*K*_a_ ∼ 8.3) compared to GSH (p*K*_a_ ∼ 9).^7^ This higher p*K*_a_ and the correlated lower reactivity might explain why GSH and not Cys is the main low-molecular-weight thiol in cells. Indeed, cells spend high energetic efforts to avoid nonspecific thiol reactions, allowing better control of other metabolically relevant thiol reactions.

Copper and, to a lesser extent, iron are able to catalyze the oxidation of thiols by O_2_.^7^ Although the precise mechanism of Cu^II^-catalyzed thiol oxidation has not been unambiguously ascertained, it supposedly involves the formation of a thiolate–Cu^II^ (RS^–^–Cu^II^) complex accompanied by thiol deprotonation (ii), the inner-sphere electron transfer from the thiol to Cu^II^ forming Cu^I^ and a thiyl radical RS^•^ (iii), the combination of two RS^•^ radicals forming the disulfide RSSR (iv), and the reoxidation of Cu^I^ to Cu^II^ by O_2_ with the formation of a superoxide radical anion, O_2_^•–^ (v).^8^
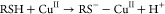
ii
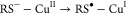
iii
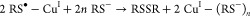
iv
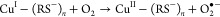
vIn alternative to reactions ii and iii, a disulfide radical anion, RS^•–^–SR, could be formed and then oxidized to the disulfide RSSR.^9^ In addition, GSSG can also be formed by reactions between GSH and O_2_^•–^ or H_2_O_2_.^10^

**Chart 2:**
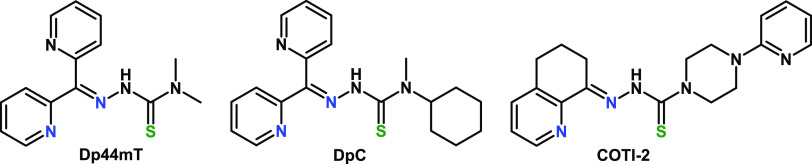
Structures of Dp44mT and Its Clinically Relevant Derivatives DpC and COTI-2

In the present combined experimental and computational study, we report on the very surprising faster GSH oxidation under aerobic conditions at lower pH together with the attempts to decipher the mechanistic aspects of the reaction. Such faster GSH oxidation is catalyzed by the Cu^II^ complex with the anticancer α-pyridyl thiosemicarbazone (TSC) Dp44mT (di-2-pyridylketone-4,4-dimethyl-3-thiosemicarbazone, see Chart 2). This observation is not only of chemical interest, as it concerns the efficient thiol oxidation at lower pH, but also has a biological and medicinal impact considering that Dp44mT is a well-investigated model compound, with two derivatives, namely, DpC and COTI-2, entering phase I clinical trials for the treatment of advanced cancer during the last few years (see Chart 2, clinical trial numbers NCT02688101 and NCT02433626, respectively).^11^ Indeed, Dp44mT, although not being clinically tested itself, benefits from higher water solubility than DpC and COTI-2 and hence has been frequently used to study the interaction of this thiosemicarbazone subtype with diverse metals with a special focus on copper and iron.^12,13^

In more detail, the studies on Dp44mT showed a pronounced synergism with Cu^II^,^11^ suggesting the involvement of Cu chelation in its mode of action,^13,14^ and Cu^II^–Dp44mT was able to reduce the cellular GSH/GSSG ratio, possibly via the generation of ROS.^15^

**Scheme 1:**
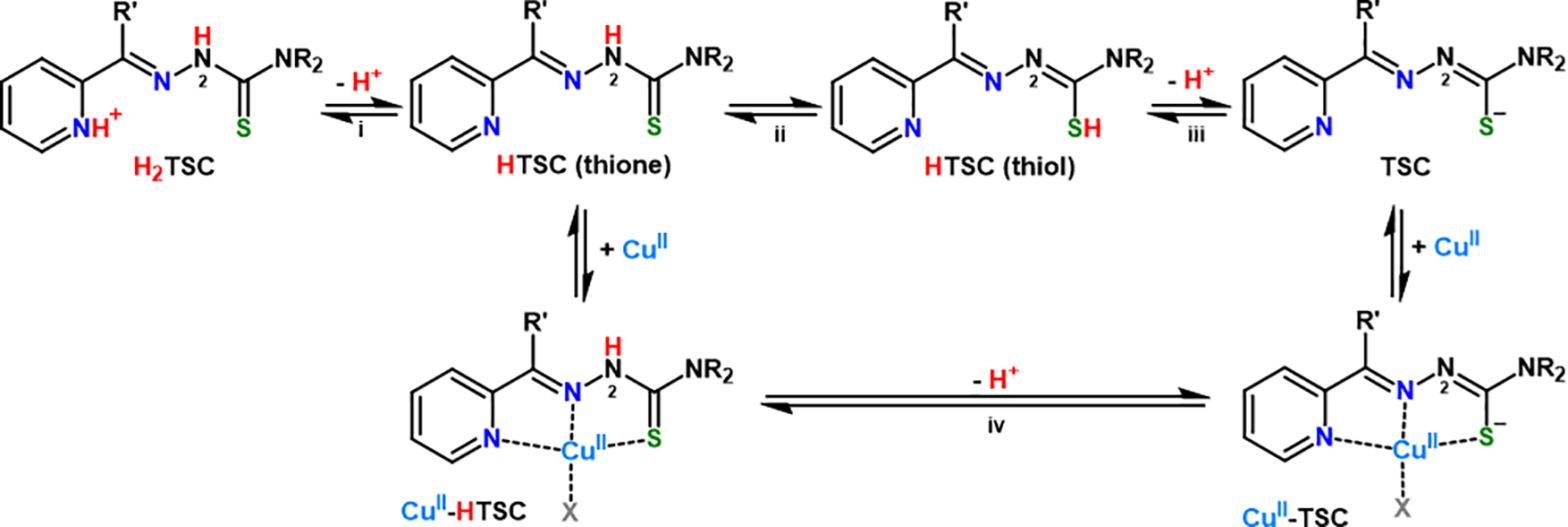
Protonation Equilibria of TSCs and Their Cu^II^ Complexes: (i) (De)protonation of α-Pyridyl Nitrogen, (ii) Thione–Thiol Tautomerism, (iii) (De)protonation of the Thioamide Moiety, and (iv) (De)protonation of Hydrazinic Nitrogen in the Cu Complex

Interestingly, Richardson and coworkers reported that Cu^II^ and Dp44mT colocalize in the lysosomes of cells treated with Cu^II^–Dp44mT, where the pH is typically ∼4.5–5.5. Notably, the partial positive charge of Dp44mT at acidic pH (see Scheme 1) was proposed to be responsible for the lysosomal accumulation of the ligand, which can enter the organelle in its neutral formal via passive diffusion or through *P*-glycoprotein. Moreover, Dp44mT showed to induce lysosomal membrane permeabilization, which is often ROS-mediated, in a Cu-dependent manner. This finding, together with the absence of high-affinity Cu proteins (e.g., metallothioneins), supports the existence of the redox-active Cu^II^–Dp44mT complex in this cell compartment.^15,16^ Besides, Cu^II^–Dp44mT is able to inhibit the ER-resident enzyme protein disulfide isomerase (PDI), which has been hence proposed as a potential target of certain Cu^II^–TSC complexes in cancer cells. Likely, Cu^II^–TSC might interfere with PDI function through the binding and/or oxidation of essential cysteine residues.^17^

α-Pyridyl TSCs are tridentate ligands forming square planar Cu^II^ complexes. Depending on the pH, two Cu-bound species may exist, namely, Cu^II^–HTSC and Cu^II^–TSC, which differ in the protonation state of noncoordinating hydrazinic (N^2^) nitrogen (see equilibrium iv in Scheme 1) and for the character of the S donor. Indeed, upon N^2^ deprotonation, thione–thiol tautomerism shifts toward the negatively charged thiolate form (see equilibrium ii, Scheme 1). Generally, such (N_py_, N, S^–^) coordination mode predominates at pH > ∼3.^12,18^

Moreover, Cu^II^–TSCs complexes have negative reduction potentials (vide infra),^19^ since the imposed square planar geometry is rather unsuited for Cu^I^ binding. As a result, Cu^II^–TSC complexes are not reduced by ascorbate even in large excess, in line with the higher redox potential of ascorbate.^14,20^ Similarly, Cu^II^–Dp44mT and its analogues, unlike other TSC complexes, are also quite resistant to the reductive dissociation by GSH under anaerobic conditions.^14^

## Results and Discussion

### Reactivity Between Cu^II^–Dp44mT and GSH at pH 7.4 vs pH 5

The interaction between Cu^II^–Dp44mT and a physiological amount of GSH (3 mM, ∼100-fold excess with respect to the complex) under aerobic conditions was first investigated via UV–vis absorption spectroscopy. Consistent with previous reports, Cu^II^–Dp44mT appeared to be resistant to the dissociation by GSH, forming a (GS^–^)–Cu^II^–Dp44mT ternary complex, as indicated by the steady red-shifted (from ∼412 to ∼416 nm) S → Cu^II^ charge transfer (CT) absorption band (see Figure S1A).^21^ We further confirmed the formation of such a ternary complex using experimental and simulated Raman spectra (see Figure S2), where the ν(Cu–N) vibration, as predicted by simulations, downshifts from 551 cm^–1^ for Cu^II^–Dp44mT to 546 cm^–1^ upon GS^–^ binding.

However, despite the apparent stability of this ternary complex over time, we observed a gradual increase in the absorbance at ∼254 nm (see Figure S1A). This band may arise from the formation of glutathione disulfide (GSSG) upon transient Cu^II^ reduction by GSH and reoxidation by O_2_, forming ROS such as O_2_^•–^, H_2_O_2_, and HO^•^ (see Figure S1C and reactions vi–ix)
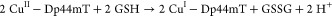
vi
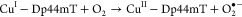
vii
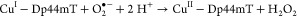
viii
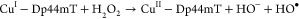
ixIndeed, the observed absorbance increase roughly matches the conversion of 3 mM GSH to 1.5 mM GSSG (ε_248_ = 380 M^–1^·cm^–1^).^22^ To assess whether the absorbance increase at 254 nm corresponds to GSH oxidation, we monitored the reaction by high-performance liquid chromatography (HPLC), confirming the formation of GSSG and the consumption of GSH (see Figure S3A). The good correlation between the spectral change at 254 nm and the HPLC peak area corroborated the attribution of the band at 254 nm to GSSG (see Figure S4). Of note, spectroscopic and HPLC analysis also showed that Cu^II^–Dp44mT catalyzed GSH oxidation faster than Cu^II^ only (see Figure S5).

**Figure 1:**
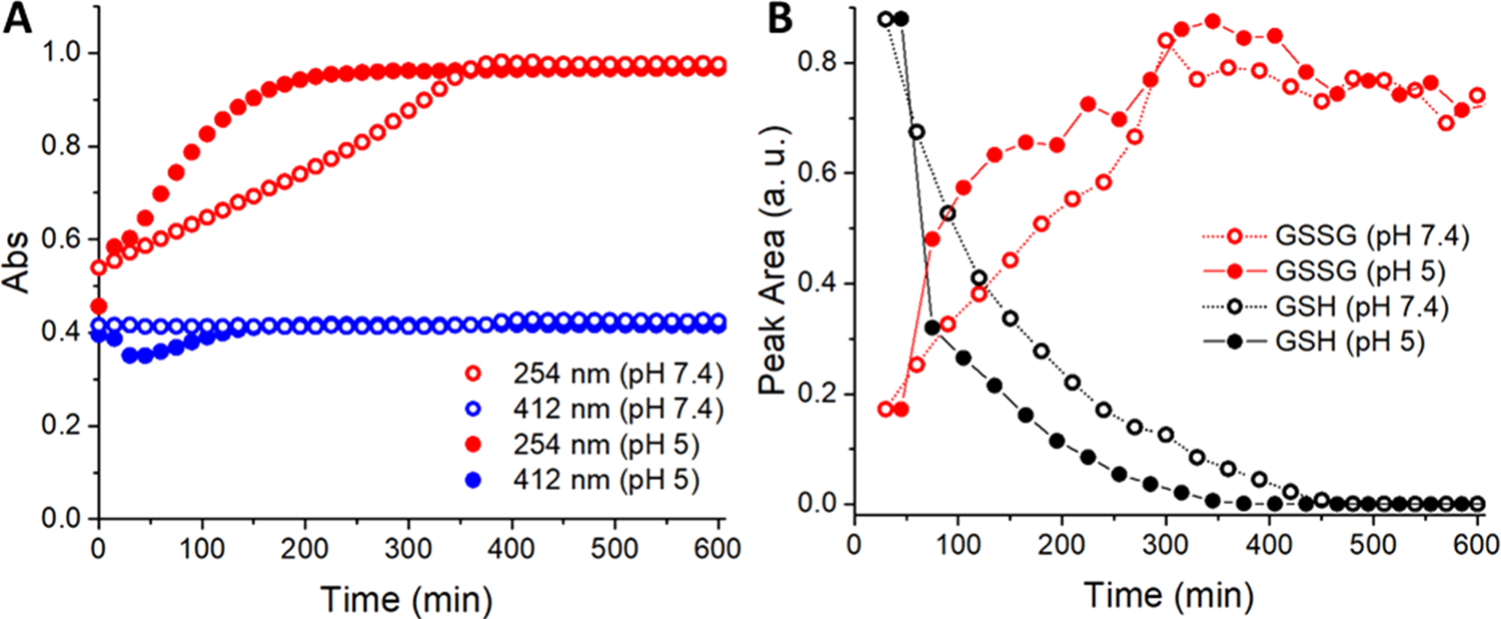
(A) Absorbance changes at 254 nm (red) and 412 nm (blue) upon the interaction of Cu^II^–Dp44mT with GSH at pH 7.4 (empty circle) and 5 (full circle). (B) GSH oxidation to GSSG followed by HPLC. Conditions: [Cu^II^] = 27 μM, [Dp44mT] = 30 μM, [GSH] = 3 mM, buffer: 100 mM HEPES pH 7.4 or 100 mM MES pH 5, and DMSO 2%.

The catalysis of GSH oxidation by Cu^II^–Dp44mT reveals that despite the apparent kinetic stability of the complex, it is redox-active, i.e., it can be reduced by GSH and reoxidized by O_2_. Indeed, reduction of the complex was observed under anaerobic conditions by monitoring the decrease of the CT band at ∼416 nm (see Figure S6). Thus, the apparent stability of the (GS^–^)–Cu^II^–Dp44mT species can be explained by faster oxidation of the [Cu^I^–Dp44mT] intermediate by O_2_ (reaction vii) compared to the reduction of Cu^II^–Dp44mT by GSH (reaction vi), which hence represents the rate-limiting step. Next, owing to the coaccumulation of Dp44mT and Cu^II^ in lysosomes observed by Richardson and coworkers,^15,16^ we also investigated the interaction of Cu^II^–Dp44mT and GSH at pH 5. Raman spectra confirmed the binding of GSH to Cu^II^–Dp44mT at pH 5 (see Figure S2C). The time-dependent UV–vis spectra of the mixture showed the partial and transient decrease of the CT band at ∼412 nm and the concurrent increase of the absorbance at 254 nm (see Figure S1B). Surprisingly, such an increase was faster at pH 5 than that at pH 7.4 (see Figure 1A). Likewise, HPLC analysis revealed that GSH oxidation to GSSG was faster at pH 5 than that at pH 7.4. (see Figures 1B and S3B).

**Figure 2:**
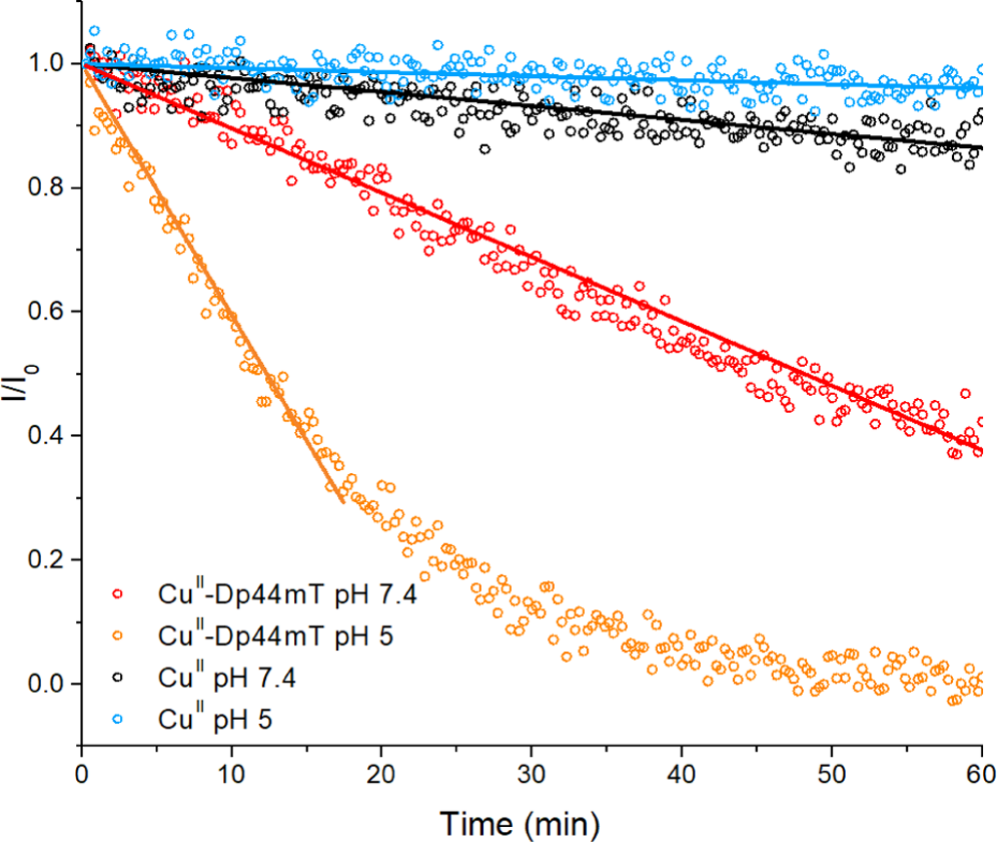
Decay of the TEMPOL EPR signal in the presence of Cu^II^–Dp44mT or Cu^II^ and GSH at pH 7.4 or 5. Conditions: [Cu^II^] = 27 μM, [Dp44mT] = 30 μM, [GSH] = 3 mM, [TEMPOL]_0_ = *I*_0_ = 20 μM, buffer: 100 mM HEPES pH 7.4 or 100 mM MES pH 5, and DMSO 2% (in the presence of Dp44mT). The initial decay of TEMPOL EPR intensity (solid lines) was linearly fitted to estimate the HO^•^ production rate (slope of the fitted curves).

Besides, as Cu^II^–Dp44mT was previously shown to produce ROS in the presence of thiols such as cysteine,^15,16^ we evaluated the generation of the HO^•^ radicals in the presence of Cu^II^–Dp44mT and GSH via electron paramagnetic resonance (EPR) spectroscopy, using TEMPOL (4-hydroxy-2,2,6,6-tetramethylpiperidin-1-oxyl) as a radical scavenger (the EPR signal of the stable nitroxyl TEMPOL radical is quenched upon reaction with radicals such as HO^•^).^23^ Thus, we also observed a ∼ 4-fold faster HO^•^ production at pH 5 than that at pH 7.4 (see Figure 2).

In contrast, the Cu^II^-catalyzed HO^•^ production and GSH oxidation in the absence of an added Cu^II^ ligand are slowed down at lower pH (see Figures 2 and S7).^24^

In light of the fact that the reduction of the complex is rate-limiting, we assessed whether this step was affected by the pH variation. Indeed, the reduction of Cu^II^–Dp44mT under anaerobic conditions appeared to be faster at lower pH (see Figure S6).

Furthermore, cyclic voltammetry (CV) measurements (see Figure S8) performed in 25% aqueous DMSO showed that the midpoint potential of the Cu^II^–Dp44mT complex is higher at pH 5 (−6.5 mV vs SHE) than that at pH 7.4 (−52.5 mV vs SHE). In addition, the comparison of the peak-to-peak separation (−163 mV at pH 7.4 vs −73 mV at pH 5) and of the anodic/cathodic peak current ratio (∼1.3 at pH 7.4 vs ∼1.08 at pH 5) revealed higher reversibility at lower pH. Of note, this results from a shift of the cathodic peak, while the anodic peak does not appear to be pH-dependent. Hence, CV experiments confirmed that the reduction and redox cycling of the complex are easier at lower pH.

Such observations appear very surprising and puzzling, since, as mentioned above, the rate of thiol oxidation is normally much slower at lower pH due to the lower reactivity of thiol compared to thiolate. Hence, we posit that different pH-dependent speciation of the Cu^II^–Dp44mT complex, rather than the thiol reactivity, is accountable for the unusual pH-dependent behavior observed.

### DFT Calculations

**Scheme 2:**
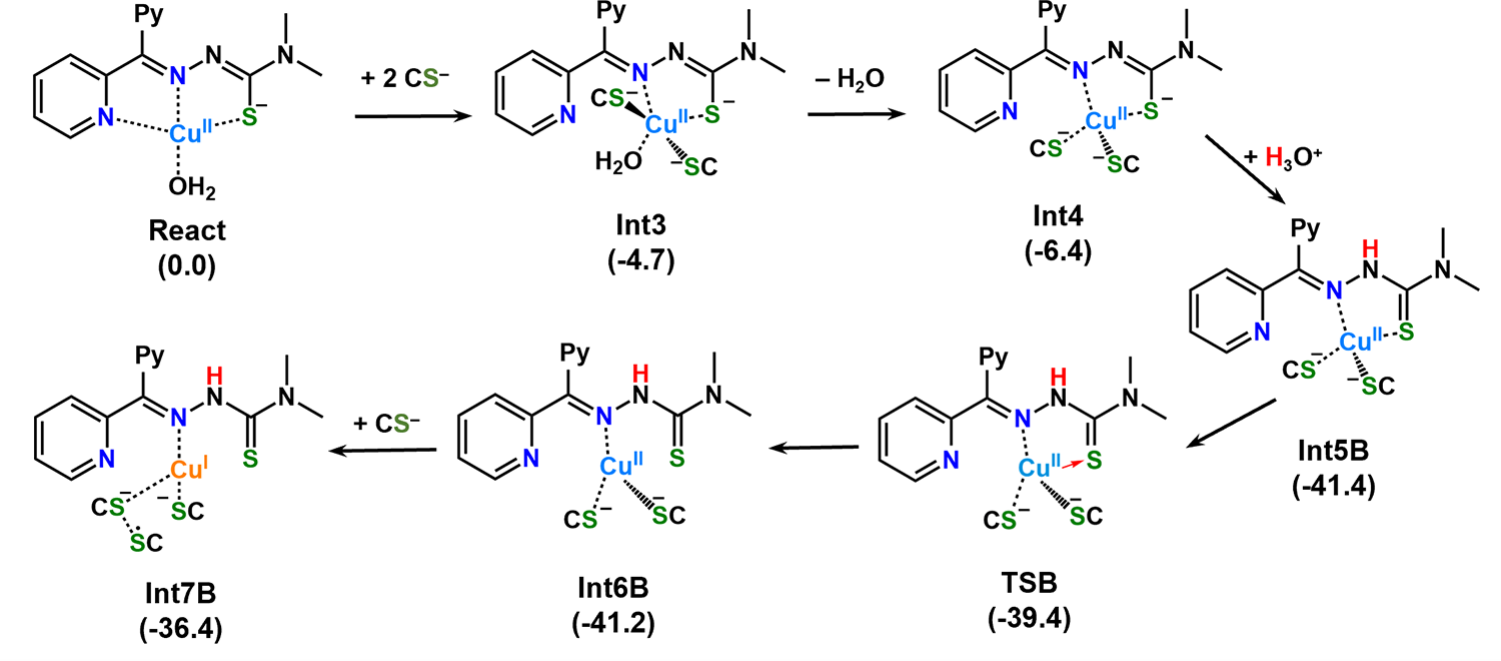
Main Steps of the DFT-Calculated Mechanism of Cu^II^–Dp44mT Reduction in the Presence of Three Deprotonated Cysteines The relative Gibbs free energies (Δ*G*298 K) are given in brackets in kcal·mol^–1^. In the transition state (**TSB** connecting the minima **Int5B** and **Int6B**), a red arrow represents the detachment of the ligand S from the Cu ion.

To investigate the mechanism of the reaction between Cu^II^–Dp44mT and GSH and in an attempt to rationalize the unexpected pH-dependent behavior, we performed quantum mechanical density functional theory (DFT) calculations. The sequence of steps that leads to Cu^II^–Dp44mT reduction by GSH was investigated using l-cysteine (Cys) as a thiol model to reduce the required computational efforts. On the basis of the experimental findings showing that at physiological pH, the Dp44mT ligand is deprotonated on the hydrazinic N^2^ atom (see Scheme 1) when coordinated to Cu^II^,^14^ calculations were carried out considering the complex in its deprotonated form Cu^II^–Dp44mT with a water molecule occupying the fourth position of the nearly square planar geometry (see **React** in Scheme 2). The main steps describing the mechanism of the reaction are reported in Scheme 2. The sum of the energies of the starting Cu^II^–(Dp44mT)(H_2_O) complex, three deprotonated cysteines (Cys^–^), and a H_3_O^+^ unit (see below) was set as the reference zero energy for calculating relative Gibbs free energies, Δ*G*^298 K^. Fully optimized geometrical structures of the located stationary points are collected in Figure S9. The first adduct, **Int1**, formed between Cu^II^–Dp44mT and one approaching Cys^–^, which is more stable than the separated reactants by 10.4 kcal·mol^–1^, is characterized by the electrostatic interaction between both thiol sulfur and one of the carboxylate oxygen atoms with water hydrogens (see Figure S9).

One of the two other Cys^–^ coordinates to Cu, forming the **Int2** adduct, only slightly more stable, by 1.3 kcal·mol^–1^, than the previous one (see Figure S9). Owing to the coordination of the second Cys^–^, a pseudo square pyramidal structure is adopted by the complex **Int3** (see Scheme 2 and Figure S9), lying below the reference energy of the separated reactants by 4.7 kcal·mol^–1^.

Simultaneously, the bonds with pyridine nitrogen N_py_ and the water molecule elongate preluding to their definitive detachment that occurs in the next minimum **Int4**, accompanied by further stabilization of 1.7 kcal·mol^–1^ (see Scheme 2 and Figure S9). All these reorganizations occurring in the presence of Cys^–^ units do not involve any electron transfer. **Int4** adopts a pseudo-tetrahedral geometry, and the Dp44mT ligand continues to be firmly bound to the copper center in a bidentate fashion through the N and S atoms, even if the bond with the ligand S atom is longer than in the tridentate coordination (see Scheme 2). It is also noteworthy that the third Cys^–^ does not get involved in any interaction with the Cu center. All of the attempts to trigger a rearrangement leading to the reduction of Cu^II^ to Cu^I^ failed, as we proved by performing a spin density analysis. The latter showed that the reduction was only accomplished by manually detaching the ligand from Cu, leading to the **Int5A** product (see Figures S9 and S10), whose formation is thermodynamically disfavored (less stable than the separated reactants by 8.8 kcal·mol^–1^) and is not connected to the preceding minimum through either a spontaneous reorganization or a transition state.

Motivated by the outcomes of preliminary calculations performed for the protonated form of the complex Cu^II^–HDp44mT and by the hypothesis formulated on the basis of the experimental findings illustrated above, we have explored the possibility that the Cu reduction is driven by the reprotonation of hydrazinic N^2^ nitrogen (see Scheme 2). Indeed, N^2^ protonation shifts the character of the S donor from thiolate to weaker thione (see equilibrium ii in Scheme 1) and hence may favor S decoordination, forming a nonplanar intermediate more prone to Cu^II^ reduction. Interestingly, the importance of such partial decoordination on the reduction of Cu^II^ by GSH was recently shown with the tridentate peptide ligand GHK.^25^ It is also worth mentioning that the change in charge from the negative thiolate to a neutral thione is expected to decrease the electron density on Cu^II^ and hence favor reduction to Cu^I^.

**Figure 3:**
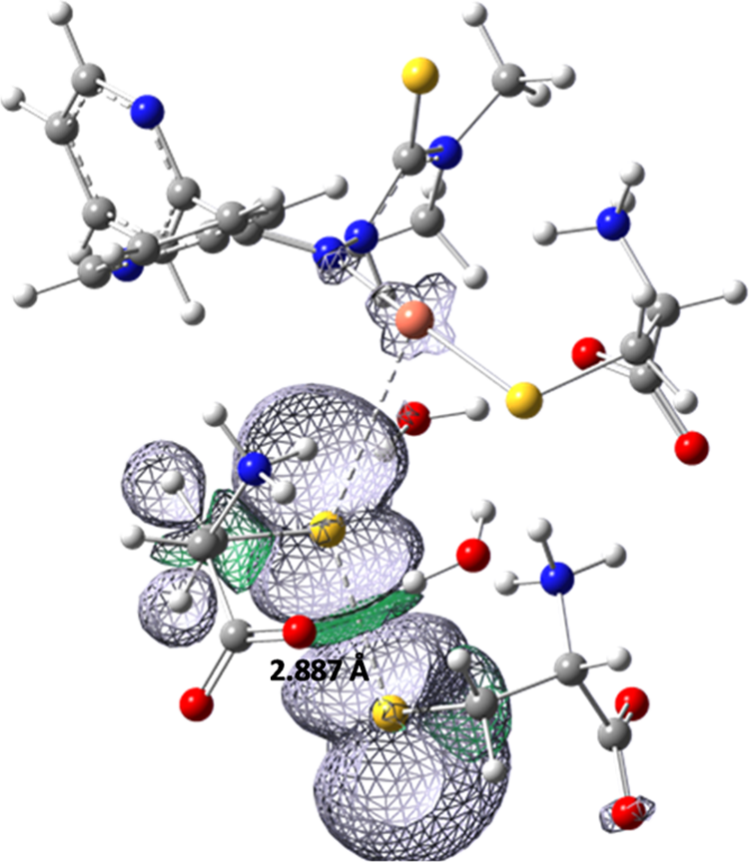
Electronic spin density plot for the product **Int7B**. The value of the calculated S–S distance is also reported.

Thus, considering that the Cu^II^–Dp44mT complex can exist in equilibrium with its protonated form Cu^II^–HDp44mT in solution (see Scheme 1), we have simulated the reprotonation of the N^2^ atom (see Scheme 1) using the hydronium ion as a protonating agent. As shown in Scheme 2, the transfer of a proton from the H_3_O^+^ unit to the N^2^ atom of the ligand leads to the formation of the new optimized minimum (**Int5B**) with a release of 41.4 kcal·mol^–1^ with respect to the zero reference energy of separated reacting species, while the formed water molecule establishes a hydrogen bond with the transferred proton. No other significant reorganization of the complex molecular structure takes place. In the effort to find a path leading to the formation of a Cu^I^ species, the very numerous used computational strategies converged on a transition state, **TSB** in Scheme 2, lying 39.4 kcal·mol^–1^ below the reference zero energy. Overcoming the energy barrier associated with the transition state **TSB** allows the definitive detachment of the S atom of the Dp44mT ligand S atom. Formation of the next connected intermediate **Int6B**, having a trigonal planar geometry with three ligands bound to the Cu center, is obtained by overcoming a **TSB** energy barrier of 2.0 kcal·mol^–1^ and is almost thermoneutral with respect to the previous minimum. Finally, the presence of the third Cys^–^ enables the reduction of Cu, obtaining a product (**Int7B**) that is less stable than the preceding minimum by only 4.8 kcal·mol^–1^. In particular, Cu^I^ results to be linearly coordinated by the iminic N atom of the HDp44mT ligand and the S atom of the equatorial Cys, while the bond with the axial Cys^–^ weakens due to the formation of a sort of adduct with the unbound Cys^–^. Indeed, as shown in Figure 3, the spin density distribution of the unpaired electron is shared by the two unbound Cys. Hence, it seems that the axial Cys^–^ acts as a bridge that allows the transfer of one electron from the unbound Cys^–^ to the Cu ion. Remarkably, the need for a third Cys to accomplish the Cu reduction, as well as the final spin density distribution, suggests that the reduction process involves the formation of a disulfide radical anion (RS^•–^–SR) rather than a thiyl radical (RS^•^) as the intermediate.^9,26^ This is also supported by the close similarity between the S–S distance in the **Int7B** adduct, namely, 2.887 Å, and the reported S–S bond length (∼2.8 Å) in disulfide radical anions.^27^ It is also worth noting that a similar disulfide radical adduct is observed in **Int5A** (see Figures S9 and S10).

Besides, it is important to underscore that in spite of the reduction of the Cu center, the Dp44mT ligand continues to be partially coordinated to the metal.^14^ This is consistent with the high stability of the complex against the dissociation by GSH and corroborates the hypothesis that the formed Cu^I^ intermediate can be easily reoxidized in the presence of O_2_. On balance, our computational analysis suggests that the protonation of hydrazinic N^2^ nitrogen is required for the reduction to occur.

### pH-Dependent Speciation of Cu^II^–Dp44mT

The hypothesis that the reduction is fostered by different pH-dependent speciation of the Cu^II^–Dp44mT complex, rather than from the reducing power of the thiol, is supported by the calculated mechanism of the reaction (Scheme 2), in which the Cu^I^ complex is formed only after the protonation of the hydrazinic N^2^ atom of the ligand.

**Figure 4:**
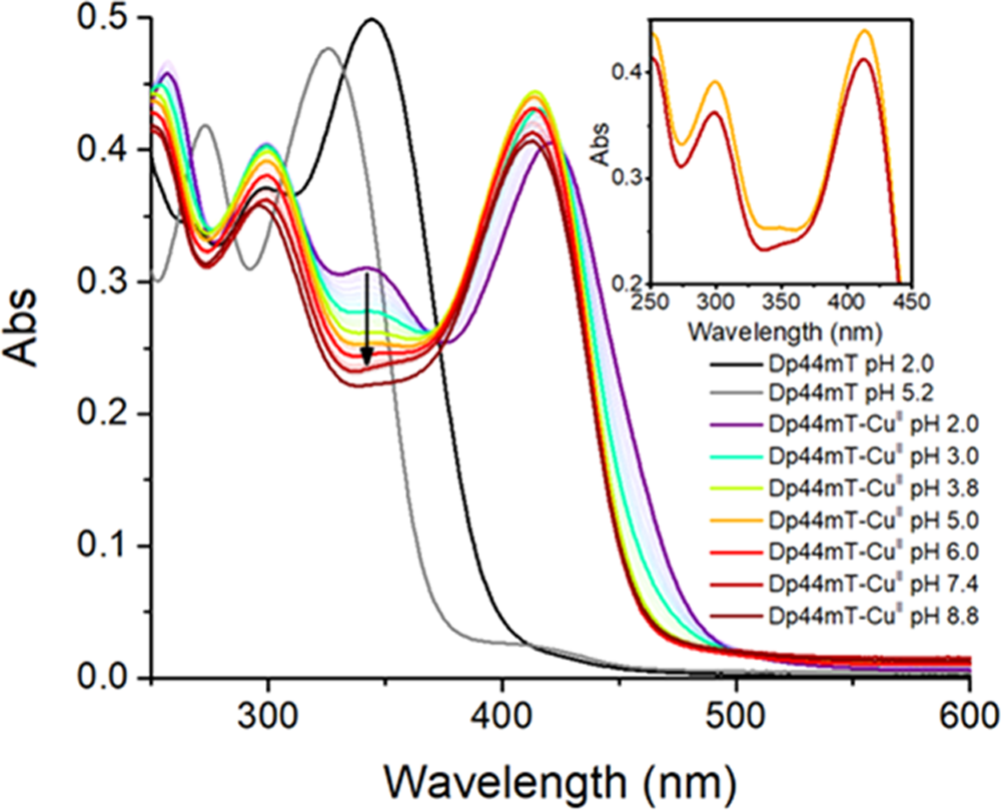
Spectrophotometric pH titration of Cu^II^–Dp44mT; inset: comparison of the spectra at pH 5 (orange) and 7.4 (red). Conditions: [Cu^II^] = 27 μM, [Dp44mT] = 30 μM, and DMSO 2%. The black arrow highlights the decrease of the band at ∼345 nm with the increasing pH.

Therefore, to obtain insights into the speciation of Cu^II^–Dp44mT as a function of pH, we performed spectrophotometric pH titration of the Cu^II^–Dp44mT complex (see Figure 4). At pH 2, a band at ∼345 nm can be clearly distinguished. Interestingly, this band decreased when increasing the pH, and hence, it can be attributed to the protonated Cu^II^–HDp44mT complex. Note that although the Cu-free doubly protonated ligand (H_2_Dp44mT, with the second proton at the noncoordinating pyridyl moiety) also absorbs at ∼344 nm, this species is absent at pH 5 (see Figures 4 and S11). In addition, we calculated the UV–vis spectra of the N^2^-protonated and -deprotonated forms of the complex using the time-dependent extension of DFT (see Figure S12). As it is evident, the peak in the computed spectrum of the protonated form of the complex appearing at 343 nm is absent in the spectrum of the deprotonated form, in good agreement with experimental spectra.

Furthermore, considering the p*K*_a_ of 2.34 previously reported for the Cu^II^–HDp44mT complex,^14^ as well as the computed extinction coefficient for the band at 343 nm (∼25 000 M^–1^·cm^–1^), we estimated the portion of N^2^-protonated Cu^II^–HDp44mT species to be as low as ∼0.2% at pH 5. In light of the proposed mechanism involving the transient protonation of the intermediate (**Int4**), such a minor yet significant portion of the N^2^-protonated form at pH 5 may account for faster GSH oxidation. Moreover, we speculate that the addition of a negatively charged GS^–^ unit to the Cu coordination sphere may increase the p*K*_a_ of hydrazinic nitrogen and hence increase the population of the N^2^-protonated species. Indeed, based on DFT calculations, the N^2^-protonation of the Cys-bound **Int4** complex results to be more favorable (by ∼12 kcal·mol^–1^) than that of the water-bound **React** species.

Besides, to assess the influence of the noncoordinating pyridyl moiety, whose p*K*_a_ is nevertheless very low (<2),^14^ we examined the behavior of some Dp44mT analogues devoid of such pyridyl group, namely, Ap44mT and PTSC (see Figure S13). These compounds also showed faster GSH oxidation at lower pH, proving that the pyridyl moiety in Dp44mT has little, if any, influence on such pH dependence (see Figure S13).

### Significance of Lysosomal pH for the Cell Toxicity of Dp44mT

**Figure 5:**
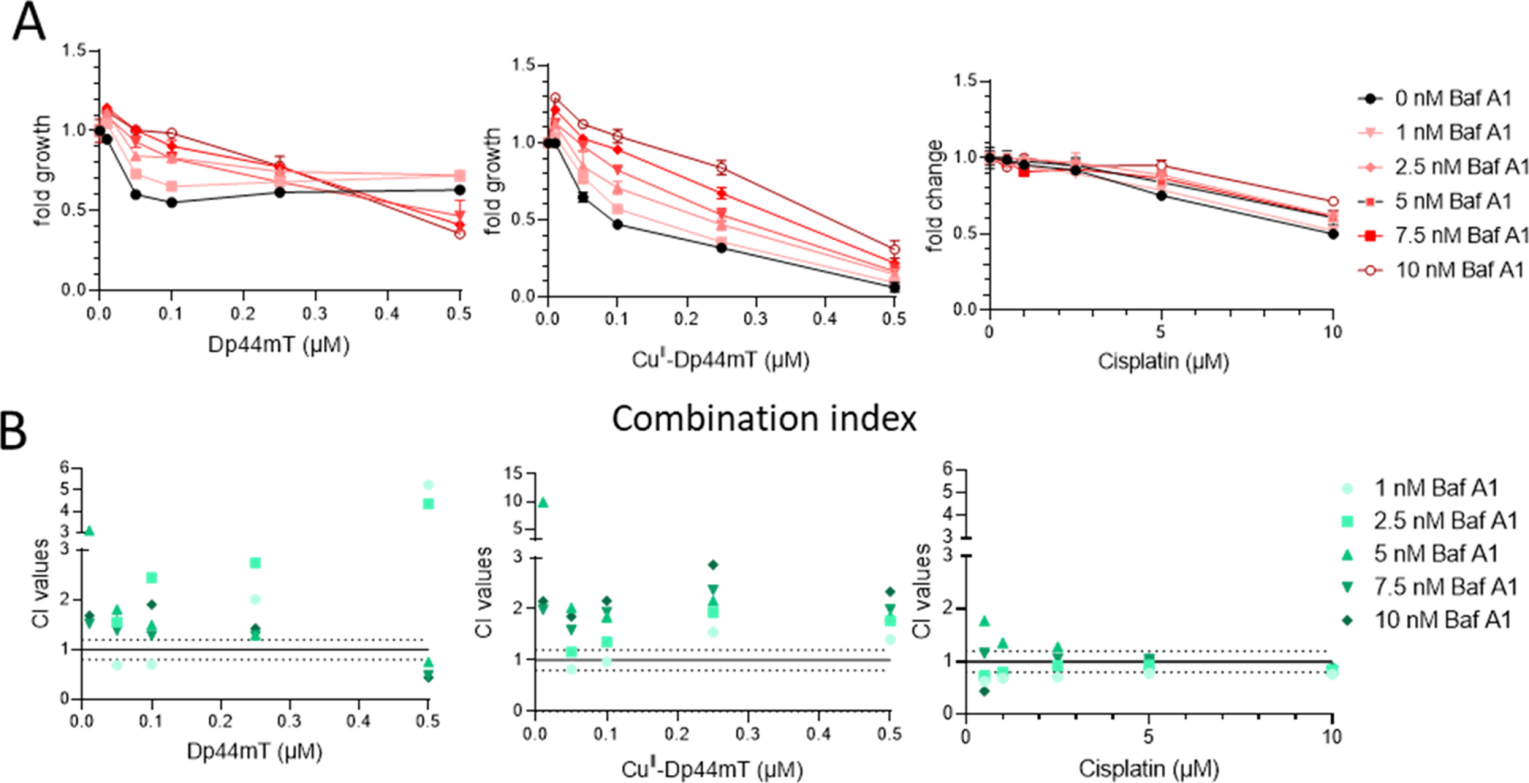
Effect of bafilomycin A1 (BafA1) on the viability of SW480 cells treated with Dp44mT, its copper complex, or cisplatin with indicated concentrations for 48 h. (A) Viability was measured by MTT viability assays. Values given are mean ± standard deviation (SD) derived from triplicates of one representative experiment out of three and normalized to cells treated with respective concentrations of BafA1 alone. (B) Combination indices were calculated by CalcuSyn. Combination (CI) values above 1 indicate antagonism, and values below 1 indicate synergism.

Based on the higher redox reactivity of Cu^II^–Dp44mT at lower pH and considering its accumulation in lysosomes (see above), we wondered if increasing the pH of the lysosomes influences the toxicity of Dp44mT. To address this question, we utilized bafilomycin A1 (BafA1), an inhibitor of the H^+^ pump responsible for the acidification of lysosomes.^28^ Briefly, SW480 cells were incubated with BafA1 for 1 h, followed by the addition of Dp44mT or its copper complex for 48 h. For comparison, the experiments were also performed with cisplatin, where no protection by BafA1 was expected. After this coincubation, cell viability was analyzed by the MTT assay, as indicated in the Experimental Section. In general, the activity of the H^+^ pump is crucial for cell functionality. Consequently, the long-term treatment with BafA1 was rather toxic to SW480 cells (∼32% at the highest concentration of BafA1, see Figure S14). However, BafA1 treatment had strong antagonistic activity (combination index values above 1) and was able to protect SW480 cells from treatment with Dp44mT or its copper complex (see Figure 5). Interestingly, these effects were more pronounced upon treatment with the copper complex than with metal-free Dp44mT. In contrast, in agreement with our hypothesis, the observed effects of BafA1 were rather minor in the case of cisplatin. These preliminary experiments indicate that, indeed, the biological activity of Cu^II^–Dp44mT is specifically promoted by the acidification of the lysosomes.

**Figure 6:**
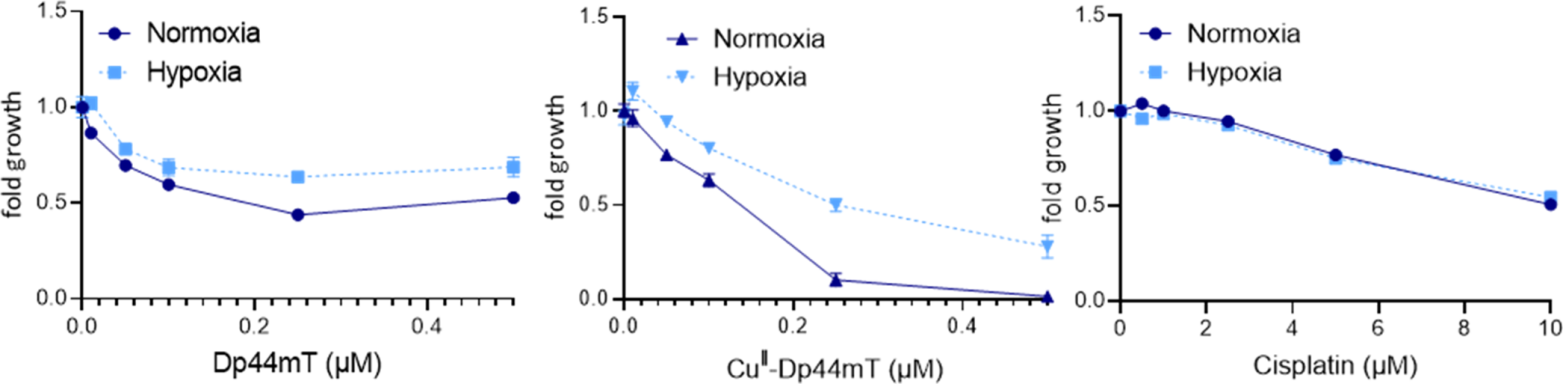
Comparison of the impact of normoxia and hypoxia on the viability of SW480 cells treated with Dp44mT, its copper complex, or cisplatin at the indicated concentrations under normoxia and hypoxia (0.1% O_2_) for 48 h. Viability was measured by MTT-based viability assays. Values given are mean ± standard deviation (SD) derived from triplicates of one representative experiment out of three and normalized to cells treated with the solvent only.

Based on the hypothesis that ROS production is involved in the lysosomal activity of Cu^II^–Dp44mT, we tested whether the drugs are less active under hypoxia. As shown in Figure 6, especially Cu^II^–Dp44mT (but to a lesser extent also metal-free Dp44mT) had visibly reduced activity in viability assays performed under 0.1% O_2_ compared to normoxic standard cell culture conditions. In contrast, no impact of hypoxia on cisplatin activity was observed. This further suggests that aerobic GSH depletion and ROS production could be involved in the cytotoxicity of Cu^II^–Dp44mT.

## Conclusions

The faster oxidation of GSH by O_2_ in the presence of Cu^II^–Dp44mT at pH 5 compared to pH 7.4 is quite remarkable. Generally, the reactivity of thiols slows down with the decrease of pH because deprotonated thiolates are the most reactive form. Indeed, GSH oxidation by Cu^II^ only, already slower than Cu^II^–Dp44mT at pH 7.4, further decelerates at pH 5. Hence, the catalytic activity of Cu^II^–Dp44mT compared to Cu^II^ only is even more remarkable at pH 5. GSH reduces Cu^II^ rapidly to form Cu^I^*_n_*(GS^–^)*_m_* clusters,^29^ whose reoxidation by O_2_ is slow and rate-limiting.^30^ In contrast, in line with the square planar coordination via five-membered chelate rings favoring Cu^II^, the reduction seems to be the rate-limiting step for Cu^II^–Dp44mT. Accordingly, an important feature for the efficient GSH oxidation in the case of Cu^II^–Dp44mT is to withstand the dissociation by GSH, which has a quite high affinity for Cu^I^,^29^ via transient coordination of Dp44mT to Cu^I^. Based on our combined spectroscopic and computational investigations, we posit that the acceleration of Cu^II^–Dp44mT reduction by GSH at lower pH is due to a higher population of the N^2^-protonated complex at low pH. This protonation lowers the affinity to Cu^II^, facilitates the reduction to Cu^I^ via partial decoordination, and hence accelerates the rate-limiting step.

The ability of Cu^II^–Dp44mT to catalyze GSH oxidation at lower pH is not only intriguing and quite exceptional from the chemical point of view but also can be of biological and medicinal interest. Indeed, Dp44mT and its analogues are well-studied anticancer agents that seem to accumulate in the lysosome as the Cu^II^–Dp44mT complex and induce lysosomal membrane permeabilization, leading to cell death. Lysosomes have a low pH (∼4.5–5.5) and contain thiols, which are needed to reduce the disulfide bonds of the proteins to digest. There are also no constitutional proteins with high Cu affinity known that could compete Cu out of Dp44mT in the lysosome.^31^ Hence, the lysosomal environment would be very favorable for fast thiol oxidation and concomitant ROS production. Here, we showed that the impairment of lysosomal acidification and hypoxia counteract the cytotoxic activity of Cu^II^–Dp44mT, suggesting that the catalysis of thiol oxidation and ROS production in lysosomes may play a role in its mode of action.

## Experimental Section

### Materials

All solvents and reagents obtained from commercial suppliers were used without further purification. TSCs were prepared as previously reported.^32^

### Preparation of Stock Solutions and Reaction Mixtures

TSC stock solutions were prepared in DMSO, and their concentration was verified via spectrophotometric Cu^II^ titrations. The Cu^II^ stock solution was prepared by dissolving CuCl_2_·2H_2_O in ultrapure water (ρ = 18.2 MΩ·cm^–1^), and its concentration was verified by UV–vis spectroscopy (ε_780_ = 12 M^–1^·cm^–1^). A stock solution of HEPES buffer (500 mM, pH 7.4) was prepared by dissolving the free acid powder in ultrapure water and adjusting the pH with NaOH. A stock solution of MES buffer (500 mM, pH 5) was prepared by dissolving MES sodium salt in ultrapure water and adjusting the pH with HCl. GSH stock solutions were prepared in ultrapure water on a daily basis. The TEMPOL stock solution was prepared in ultrapure water. The Cu^II^–TSC complexes were generated in situ by mixing a TSC solution and a CuCl_2_·2H_2_O solution in buffer. A small volume (few μL) of a GSH solution was then added to initiate the reaction.

### UV–vis Spectroscopy

UV–vis spectra were recorded in 1 cm path quartz cuvettes using an Agilent Cary 60 spectrophotometer. For the anaerobic reduction, solutions were thoroughly degassed under N_2_ before and after the insertion into a sealable cuvette equipped with a pierceable septum, through which GSH was added with a microsyringe. pH titrations were conducted by adding small aliquots of NaOH solutions to the ligand/complex solution in HCl (∼0.01 M). GSH oxidation by Cu^II^–Ap44mT/PTSC complexes was monitored via the absorbance change at 254 nm using a ClarioStar plate reader inside a microwell plate.

### HPLC and LC Mass Spectrometry (LC-MS)

The HPLC analysis of GSH and GSSG was performed using a Hitachi Primaide instrument on a C18 column (XBridge Peptide BEH C18 column from Waters, 4.6 mm × 150 mm, pore size 300 Å, particle size 3.5 μm) using 0.1% aqueous TFA (solvent A) and 90% CH_3_CN/0.1% TFA in water (solvent B) with a linear gradient from 5 to 10% solvent B in 7 min. The attribution of the peaks was achieved by comparison with a solution containing GSH or GSSG only and via LC-MS spectra that were recorded using an LCQ Fleet ion trap mass spectrometer (Thermo Fischer) coupled to a Ultimate3000 RSLCnano system equipped with an ACQUITY UPLC BEH C18 column (130 Å, 1.7 μm, 1.0 mm × 150 mm).

### Raman Spectroscopy

Raman spectra were recorded on a Renishaw inVia Raman microscope equipped with a CCD (charge-coupled device) detector. We used the 457 nm line of an argon laser focused on the sample solution with a 20× objective. Ten accumulations were averaged with an exposure time of 10 s for each sample. The collected data are smoothed with a 13-point Savitzky–Golay second-order polynomial function.

### EPR Spin Scavenging

EPR spin scavenging experiments were performed at room temperature (*T* = 295 ± 1 K) using an EMX-plus (Bruker Biospin GmbH, Germany) X-band EPR spectrometer equipped with a high sensitivity resonator (4119HS-W1, Bruker). The g factor was calibrated in the experimental conditions using the Bruker strong pitch (*g* = 2.0028). The samples were introduced into glass capillaries (Hirschmann, 25 μL) sealed at both the ends and rapidly transferred into the EPR cavity for measurement. The principal experimental parameters were as follows: a microwave frequency of ∼9.85 GHz, a microwave power of ∼4.5 mW, a modulation amplitude of 1 G, a time constant of ∼5 ms, and a conversion time of ∼12.5 ms. A scan (sweeping time of ∼10 s) was then acquired every 17 s to obtain the kinetics of TEMPOL reduction over time. All spectra were best simulated and the resulting simulations were doubly integrated to relatively quantify the concentration of remaining TEMPOL. Data analysis and simulations based on experimental data were performed using Xenon software (Bruker Biospin GmbH, Germany) and lab-made routines based on EasySpin toolbox under MATLAB (Mathworks) environment.^33^ The initial decay of TEMPOL EPR intensity was linearly fitted to estimate the HO^•^ production rate (slope of the fitted curves).

### Cyclic Voltammetry

Cyclic voltammetry was performed with a VersaSTAT4 potentiostat (Princeton Applied Research) using a 3 mm glassy carbon working electrode, a platinum counter electrode, and a Ag/AgCl (3 M KCl) reference electrode. The sweep rate was 0.1 V/s.

### DFT Calculations

All of the calculations were carried out by means of the Gaussian16 software package^34^ in the context of DFT and its TD-DFT extension. The hybrid meta functional used for geometry optimizations and frequency calculations was M05.^35^ Such a functional was employed because it accurately models metallic interactions.^36^ Within the frequency calculations, the number of imaginary frequencies, 0 or 1, was taken into account to confirm the nature of minima and transition states. In the case of the transition states, intrinsic reaction coordinate (IRC) calculations were performed to verify that the imaginary frequency corresponds to the proper motion along the reaction coordinate. The standard 6–311G* basis set of Pople was used for Cu, C, N, O, and H atoms, and 6-311 + G* basis set was used for S atoms. The solvation model based on density, SMD, was adopted in geometry optimizations for mimicking solvent effects using water as the solvent because it can be consistently used for any charged or uncharged solute in any solvent or liquid medium.^37^ To reduce the computational costs and to simulate the thiol-rich environment in which the reaction occurs, cysteine was used instead of GSH to explore the reaction mechanism. Relative Gibbs free energies (Δ*G*), including thermal corrections at 298.15 K, were calculated for all of the located stationary points of the path with respect to the zero reference energy, which is the sum of the energies of separated reactants.

Absorption spectra in an aqueous solution (SMD solvent model) were calculated, by performing 50 electronic excitations, through the TD-DFT approach. Several computational protocols were tested for the calculation of the UV–vis absorption spectra in water and for the calculation of Raman spectra vibrational frequencies to obtain a better agreement with the experimental counterpart. The B3LYP functional^38^ was chosen as the better performing, together with the Pople basis set already used for the geometry optimization. To take into account nonbonding interactions, Grimme dispersion correction was included using an atom pairwise additive scheme,^39^ the DFT-D3 method.

### MTT Viability Measurement

For viability measurements, SW480 human colon carcinoma cell models (obtained from ATCC) were used and cultured at 37 °C and 5% CO_2_ in MEME (Merck, M0268) medium supplemented with 10% fetal calf serum (PAA, Austria). The cells were plated (2 × 10^3^ cells/well) in 96-well plates and allowed to recover for 24 h. To determine the impact of the lysosomal pH on the drug efficacy, the cells were pretreated with 0, 1, 2.5, 5, 7.5, and 10 nM bafilomycin A1 for 1 h. After which, increasing concentrations of Dp44mT, its copper complex or cisplatin were added for 48 h. Cell viability was measured by the 3-(4,5-dimethylthiazol-2-yl)-2,5-diphenyltetrazolium bromide (MTT)-based vitality assay (EZ4U; Biomedica, Vienna, Austria). Combination indices were calculated by CalcuSyn using the Chou–Talalay method.^40^ Values above 1 indicate antagonism, and values below 1 indicate synergism. In the case of hypoxia experiments, plates were in parallel incubated after drug treatment either under standard cell culture conditions or in a hypoxia chamber (ProOx Model C21, Biospherix) in an atmosphere with reduced oxygen conditions (0.1%).

## Supporting Information Available

UV–vis and Raman spectra; chromatograms; voltammograms; three-dimensional (3D) structures of the calculated intermediates; control experiments (PDF)
