# Supplementary material for: Copper-Catalyzed Glutathione Oxidation is Accelerated by the Anticancer Thiosemicarbazone Dp44mT and Further Boosted at Lower pH
Source: J Am Chem Soc. 2022 Aug 5;144(32):14758–68. doi: 10.1021/jacs.2c05355 (PMC9389589; doi:10.1021/jacs.2c05355)
Supplement: Supplementary file 1 — ja2c05355_si_001.pdf [file ja2c05355_si_001.pdf]

Supplementary Information

Copper-catalysed glutathione oxidation is accelerated by the anticancer thiosemicarbazone Dp44mT and further boosted at lower pH

Enrico Falcone,[a,#] Alessandra G. Ritacca,[b,#] Sonja Hager,[c] Hemma Schueffl,[c] Bertrand Vileno,[a] Youssef El Khoury,[d] Petra Hellwig,[d] Christian R. Kowol,[e] Petra Heffeter,[c,*] Emilia Sicilia,[b,*] and Peter Faller[a,f,*]

[a] *Institut de Chimie (UMR 7177), University of Strasbourg – CNRS, 4 Rue Blaise Pascal, 67081 Strasbourg, France.*
[b] *Department of Chemistry and Chemical Technologies, Università della Calabria, Ponte P. Bucci, 87036 Arcavacata di Rende (CS), Italy.*
[c] *Center for Cancer Research, Medical University of Vienna, Borschkegasse 8a, 1090 Vienna, Austria*
[d] *Laboratoire de bioélectrochimie et spectroscopie, UMR 7140, CNRS, Université de Strasbourg, 4 Rue Blaise Pascal, 67081 Strasbourg, France*
[e] *Institute of Inorganic Chemistry, Faculty of Chemistry, University of Vienna, Waehringer Straße 42, 1090 Vienna, Austria.*
[f] *Institut Universitaire de France (IUF), 1 rue Descartes, 75231 Paris, France*

[#] These authors contributed equally
*corresponding authors: pfaller@unistra.fr (P.F.), emilia.sicilia@unical.it (E.S.), petra.heffeter@meduniwien.ac.at (P.H)

Contents

Figure S1. UV-vis spectra of Cu^{II}-Dp44mT reaction with GSH at pH 7.4 or 5 S2
Figure S2. Simulated and experimental Raman spectra S2
Figure S3. HPLC chromatograms S3
Figure S4. Correlation between A_{254nm} and HPLC peak area S3
Figure S5. Aerobic GSH oxidation catalyzed by Cu^{II}-Dp44mT and Cu^{II} only S4
Figure S6. Anaerobic reduction of Cu^{II}-Dp44mT by GSH at pH 7.4 or 5 S4
Figure S7. Cu^{II}-catalysed aerobic GSH oxidation at pH 7.4 or 5 S5
Figure S8. Cyclic voltammograms S5
Figure S9. Optimized geometrical structures of stationary points S6
Figure S10. Spin density of **Int5A** S7
Figure S11. Spectrophotometric pH titration of Dp44mT S7
Figure S12. Calculated UV-vis spectra of Cu^{II}-Dp44mT and Cu^{II}-HDp44mT S8
Figure S13. GSH oxidation by Ap44mT and PTSC Cu^{II}-complexes S8
Figure S14. Effect of bafilomycin A1 (BafA1) on viability of SW480 cells S8

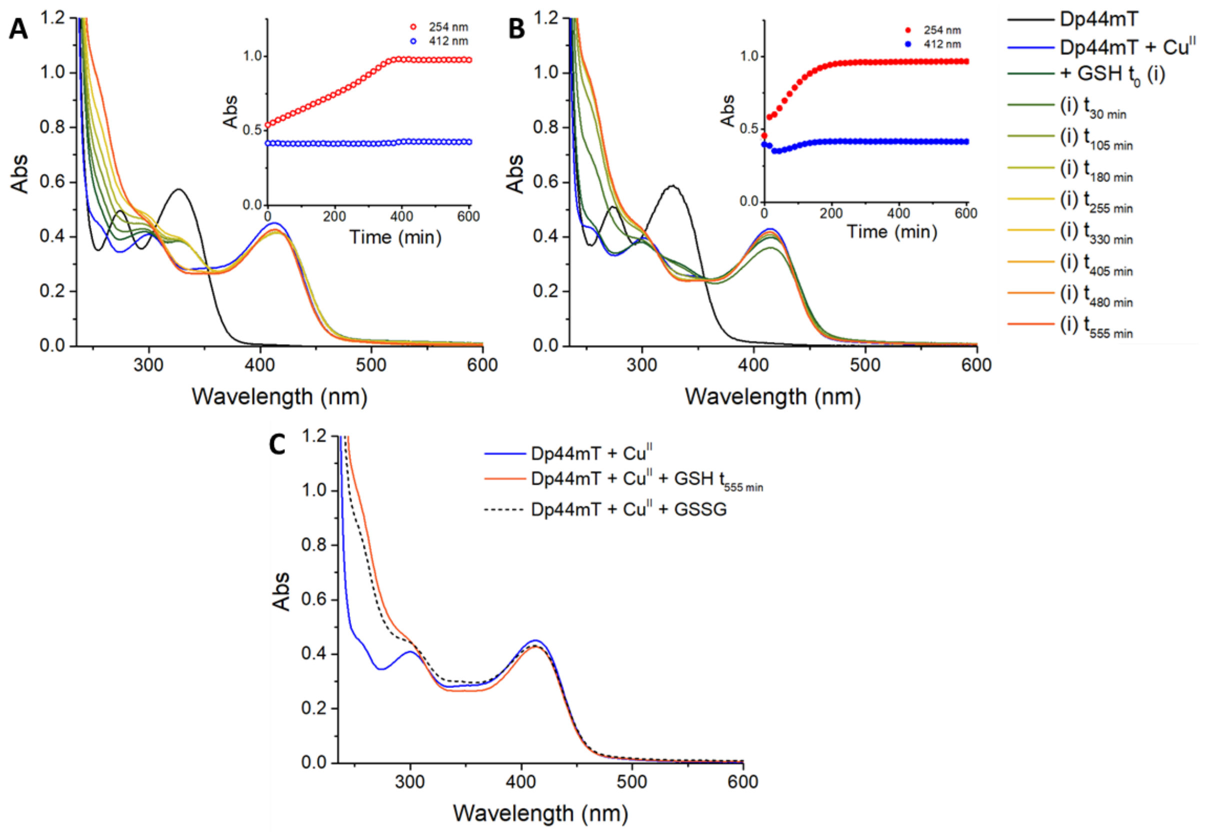

Figure S1. UV-vis spectra of Cu^{II}-Dp44mT reaction with GSH at pH 7.4 (A) and 5 (B). (C) Comparison of the final reaction mixture with a solution containing Cu^{II}-Dp44mT and GSSG. Conditions: [Cu^{II}] = 27 µM, [Dp44mT] = 30 µM, [GSH] = 3 mM (A, B), [GSSG] ~1.5 mM (C); buffer: 100 mM HEPES pH 7.4 (A) or 100 mM MES pH 5 (B); DMSO 2%. Insets show absorbance changes at 254 (red) and 412 nm (blue).

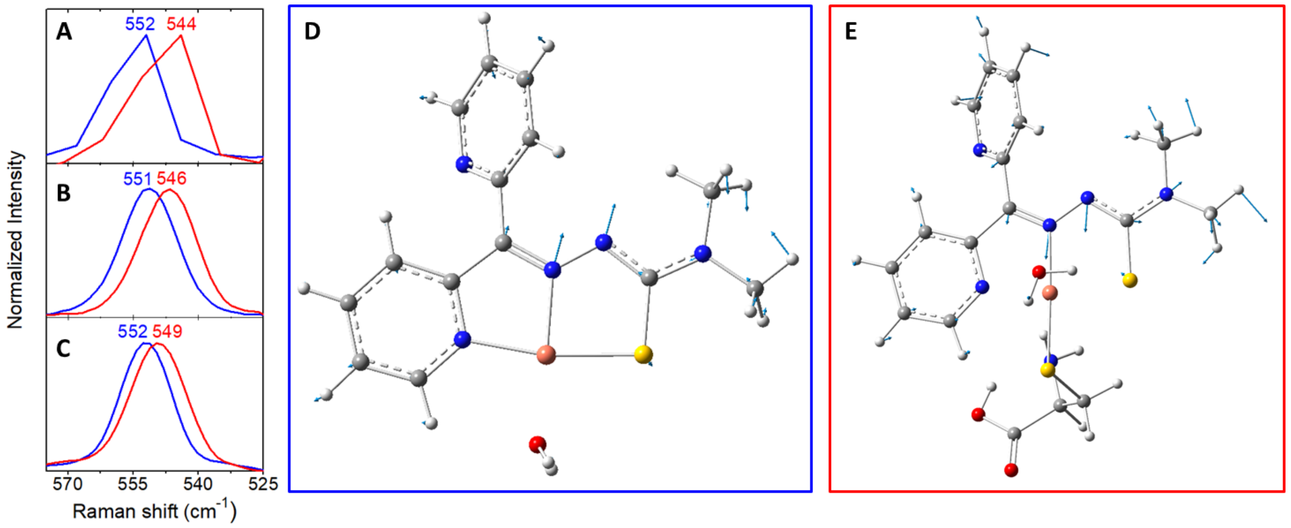

Figure S2. Simulated (A) and experimental (B, C) Raman spectra of Cu^{II}-Dp44mT (blue) and (GS^{-})-Cu^{II}-Dp44mT (red) at pH 7.4 (B) and pH 5 (C). Experimental conditions: [Cu^{II}] = 0.9 mM, [Dp44mT] = 1 mM, [GSH] = 3 mM, buffer: 100 mM HEPES pH 7.4 (B) or 100 mM MES pH 5 (C); DMSO 25%. The considered Raman frequencies are attributed to the vibrational modes shown in D and E for Cu^{II}-Dp44mT and Cys-Cu^{II}-Dp44mT, respectively.

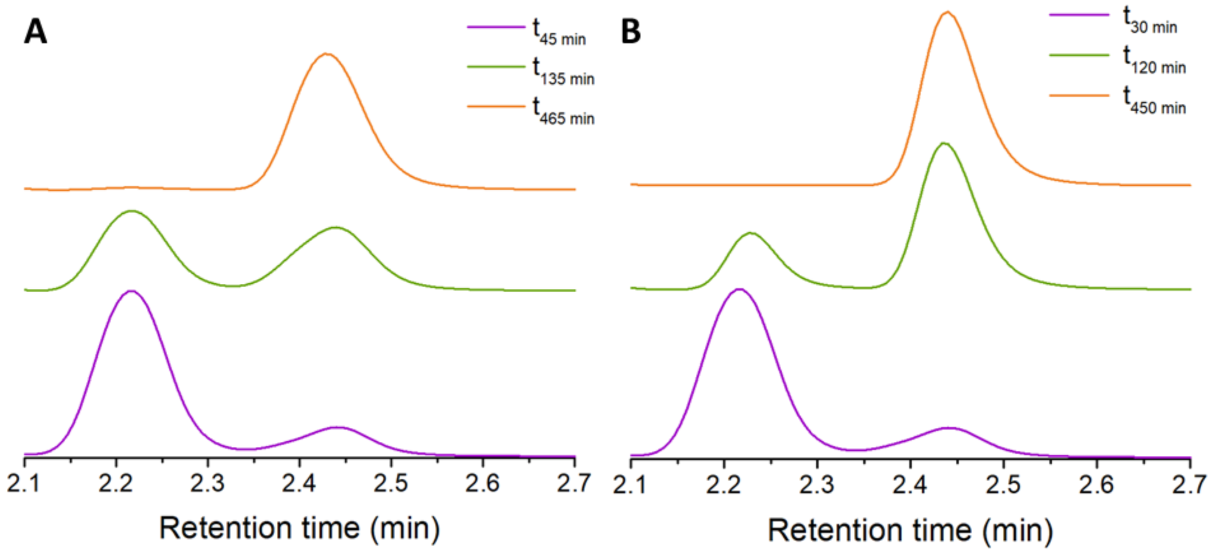

Figure S3. HPLC chromatograms showing GSH (Rt ~2.2 min) and GSSG (Rt ~2.45 min) peaks at different time points during the reaction of Cu^{II}-Dp44mT with GSH at pH 7.4 (A) and 5 (B). Conditions: [Cu^{II}] = 27 μM, [Dp44mT] = 30 μM, [GSH] = 3 mM; buffer: 100 mM HEPES pH 7.4 (A) or 100 mM MES pH 5 (B); DMSO 2%.

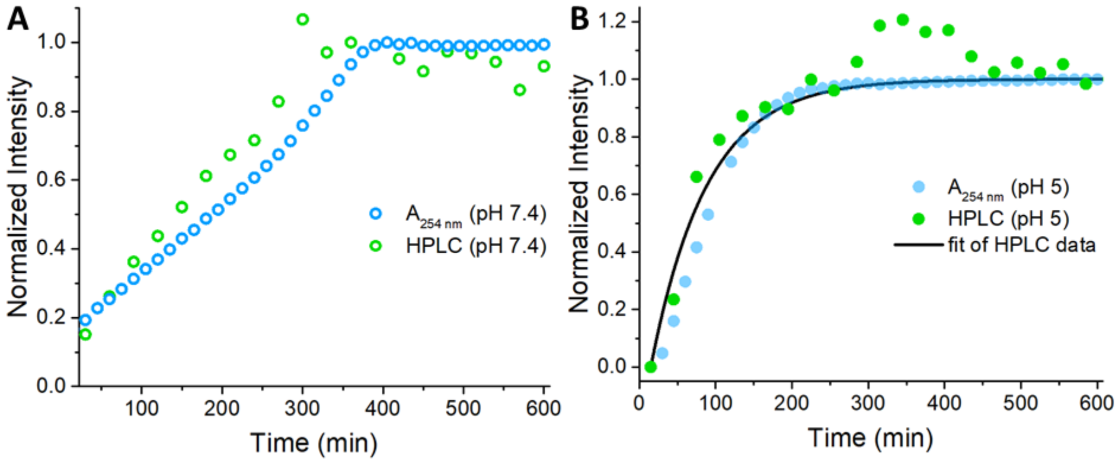

Figure S4. Correlation between the absorbance at 254 nm and GSSG HPLC peak area at pH 7.4 (A) and pH 5 (B).

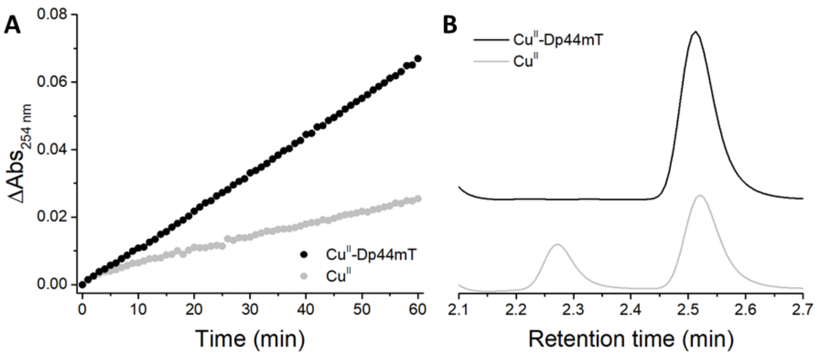

Figure S5. Comparison between the aerobic GSH oxidation catalyzed by Cu^{II}-Dp44mT (black) and Cu^{II} only (grey). (A) Absorbance changes at 254 nm; (B) HPLC chromatrograms after overnight incubation of GSH with Cu^{II}-Dp44mT or Cu^{II} only, showing complete conversion of GSH (Rt ~2.2 min) to GSSG (Rt ~2.55 min) by Cu^{II}-Dp44mT but only partial conversion by Cu^{II} only.

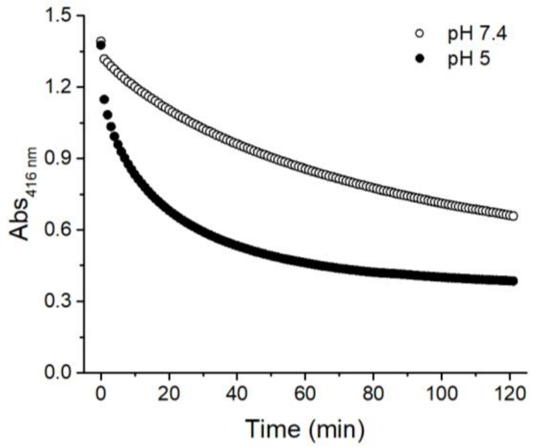

Figure S6. Anaerobic reduction of Cu^{II}-Dp44mT by GSH at pH 7.4 (empty circles) or pH 5 (full circles) monitored by UV-vis absorption spectroscopy. Conditions: [Cu^{II}] = 90 μM, [Dp44mT] = 100 μM, [GSH] = 1 mM; buffer: 100 mM HEPES pH 7.4 or MES 100 mM pH 5; DMSO ~5%.

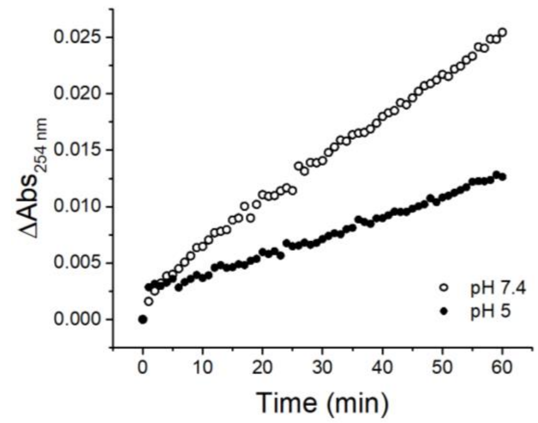

Figure S7. Cu^{II}-catalysed aerobic GSH oxidation at pH 7.4 (empty circles) or 5 (full circles). Conditions: [Cu^{II}] = 27 µM, [GSH] = 3 mM; buffer: 100 mM HEPES pH 7.4 or 100 mM MES pH 5; DMSO 2%.

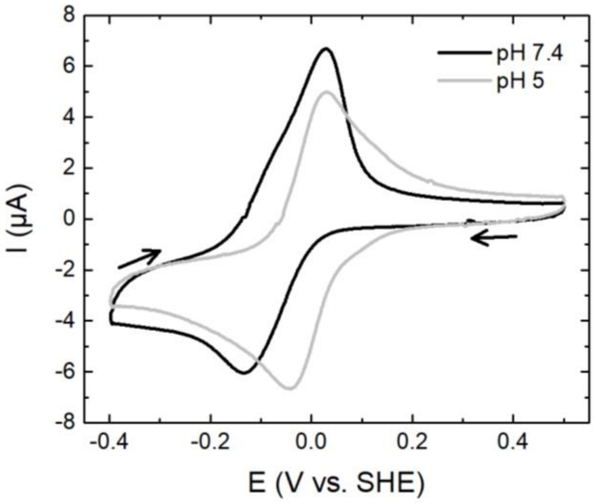

Figure S8. Cyclic voltammograms of the Cu^{II}-Dp44mT complex at pH 7.4 (black) and pH 5 (grey). The arrows indicate the scanning direction. Conditions: [Cu^{II}] = 450 µM, [Dp44mT] = 500 µM; buffer: 100 mM HEPES pH 7.4 or MES 100 mM pH 5, NaCl 100 mM, DMSO 25%. Working electrode: glassy carbon; counter electrode: Pt; Reference electrode: Ag/AgCl (3M KCl); sweep rate: 0.1 V/s. Solutions were thoroughly degassed under Ar before measurements.

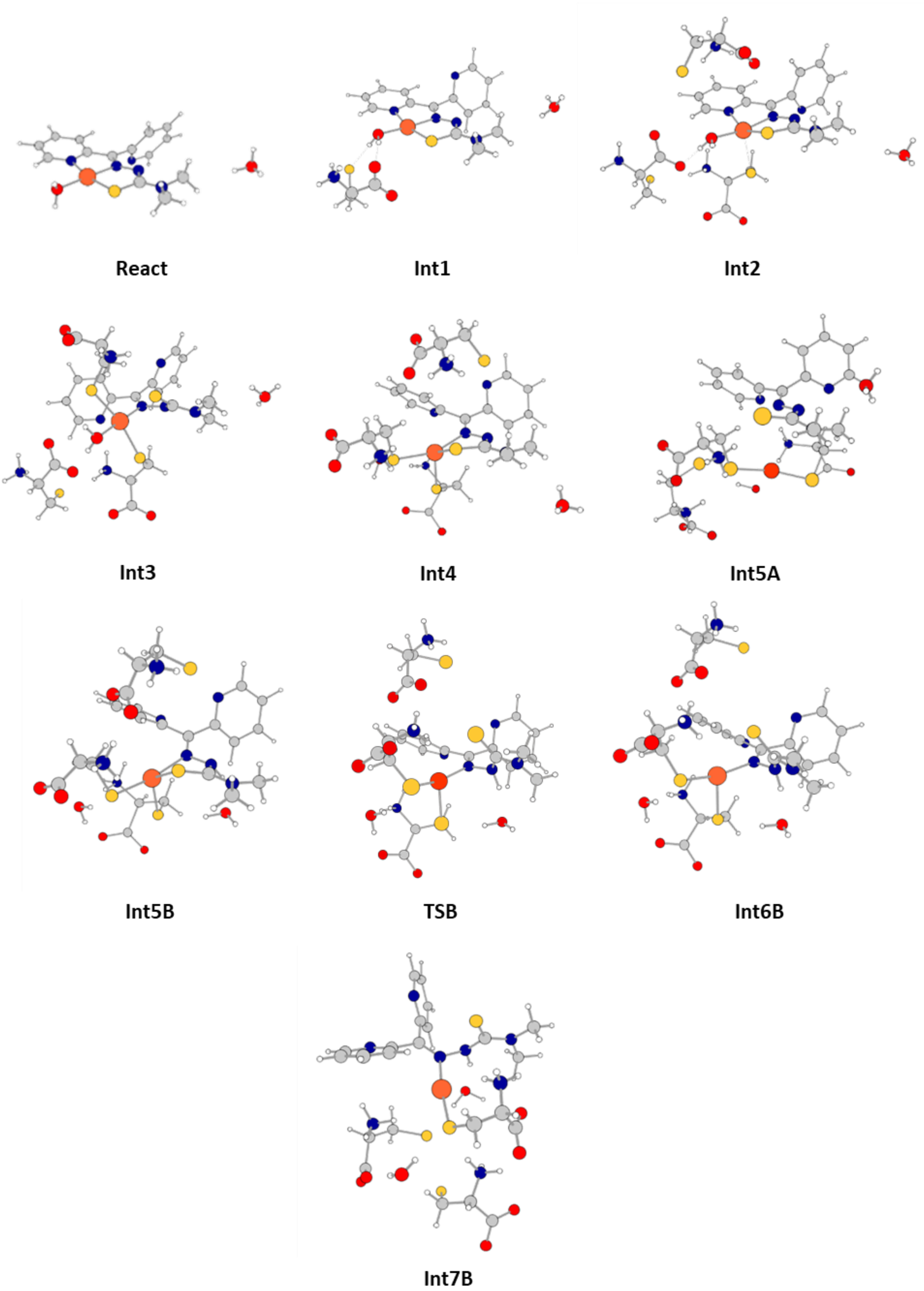

Figure S9. Fully optimized geometrical structures of stationary points describing the mechanism of the reaction between the Cu^{II}-Dp44mT complex and three deprotonated cysteines. Cu metal center is in orange, N atoms are bleu, O atoms in red and C atoms in grey.

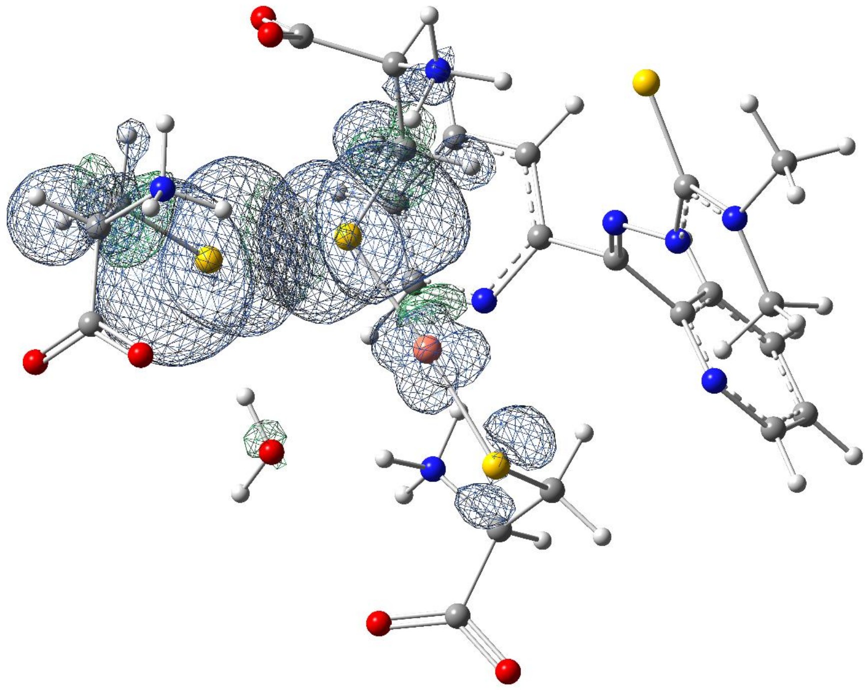

Figure S10. Spin density of **Int5A**.

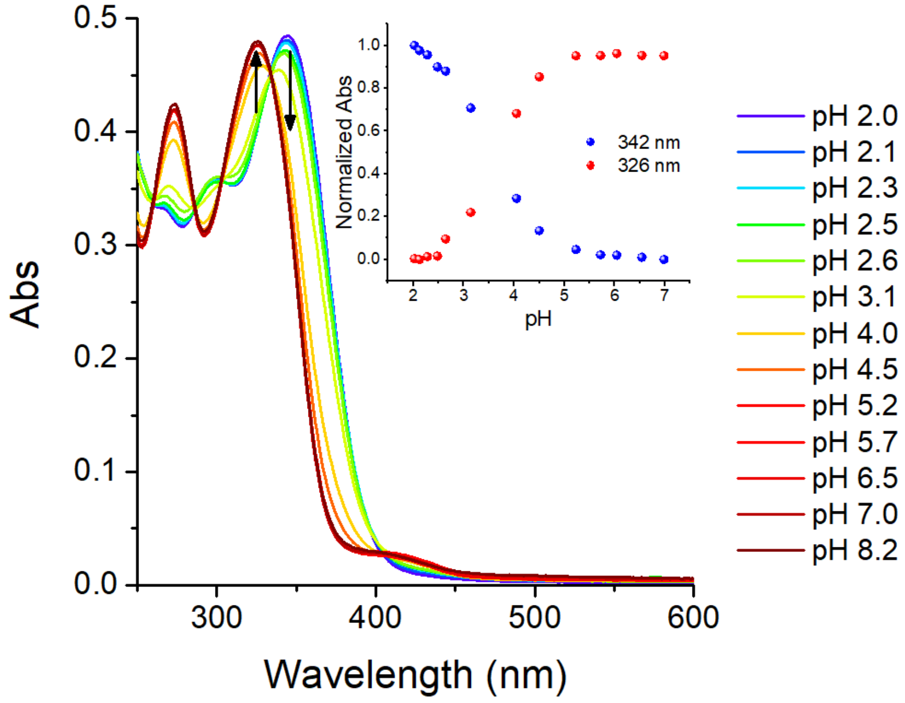

Figure S11. Spectrophotometric pH titration of Dp44mT. Conditions: [Dp44mT] = 30 µM; DMSO 2%.

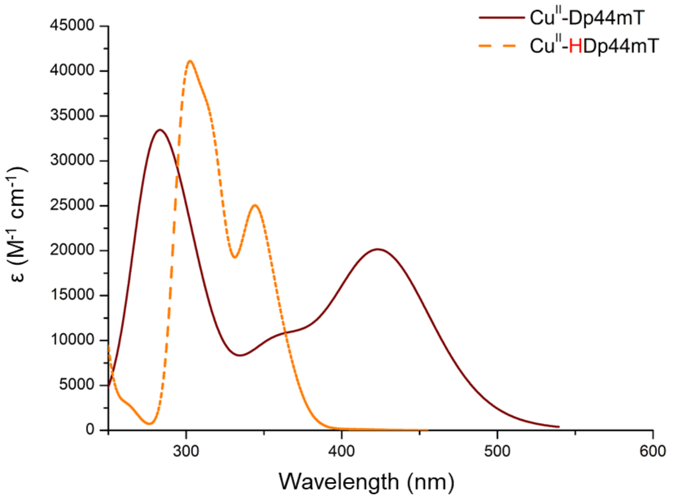

Figure S12. Calculated UV-vis spectra of the protonated and deprotonated on the N^2 atom forms of the complex.

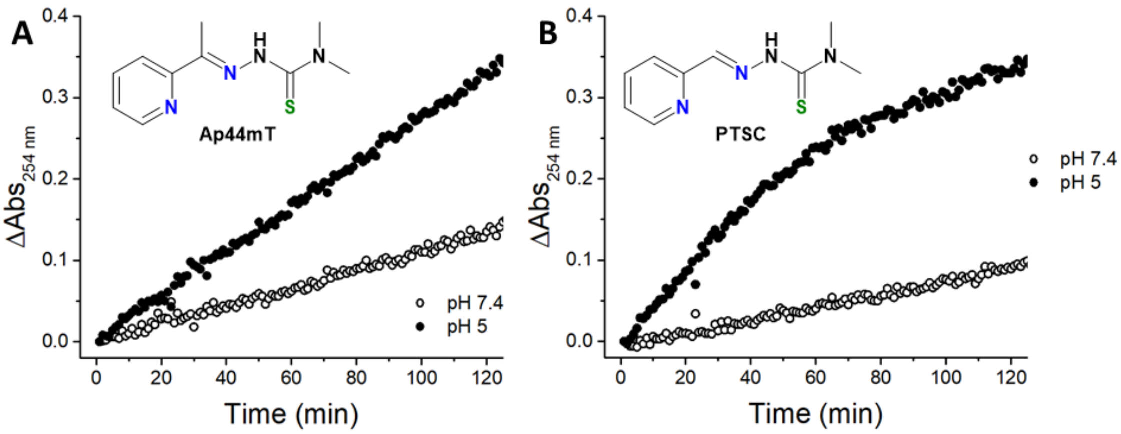

Figure S13. GSH oxidation by Ap44mT (A) and PTSC (B) Cu^{II}-complexes at pH 7.4 (empty circles) or 5 (full circles) monitored through absorbance changes at 254 nm. Conditions: [CuII] = 27 μM, [TSC] = 30 μM, [GSH] = 3 mM; buffer: 100 mM HEPES pH 7.4 (A) or 100 mM MES pH 5 (B); DMSO 2%.

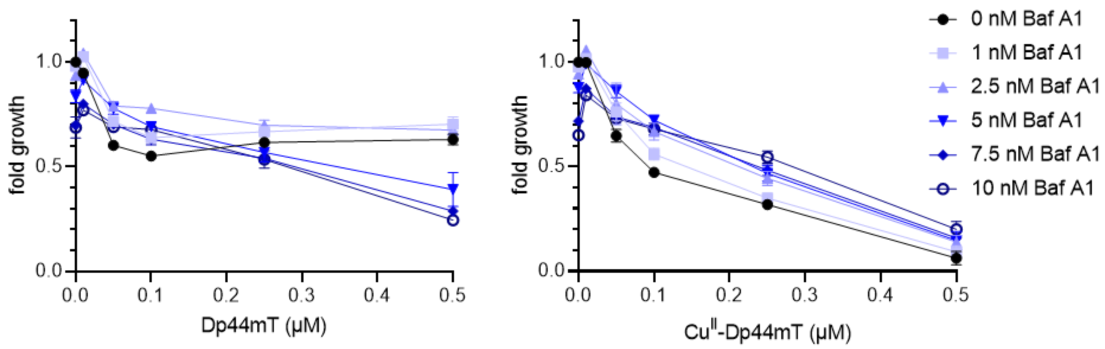

Figure S14. Effect of bafilomycin A1 (BafA1) on viability of SW480 cells treated with Dp44mT and its copper complex with indicated concentrations for 48 h. Viability was measured by MTT viability assay. Values given are the mean ± standard deviation (SD) derived from triplicates of one representative experiment out of three and normalized to untreated cells.
